# Supplementary material for: Bayesian refinement of protein structures and ensembles against SAXS data using molecular dynamics
Source: PLoS Comput Biol. 2017 Oct 18;13(10):e1005800. doi: 10.1371/journal.pcbi.1005800 (PMC5662244; doi:10.1371/journal.pcbi.1005800)
Supplement: S1 Archive — (BZ2) [file pcbi.1005800.s011.tar.bz2 › gromacs_4.62_bayesian_saxs_refi/share/html/online/g_membed.html]

g\_membed


| g\_membed Main Table of Contents |

**VERSION 4.6  
Sat 19 Jan 2013**

---

### Description

g\_membed embeds a membrane protein into an equilibrated lipid bilayer at the position
and orientation specified by the user.

SHORT MANUAL  
------------  
The user should merge the structure files of the protein and membrane (+solvent), creating a
single structure file with the protein overlapping the membrane at the desired position and
orientation. The box size is taken from the membrane structure file. The corresponding topology
files should also be merged. Consecutively, create a .tpr file (input for g\_membed) from these files,with the following options included in the .mdp file.  
- integrator = md  
- energygrp = Protein (or other group that you want to insert)  
- freezegrps = Protein  
- freezedim = Y Y Y  
- energygrp\_excl = Protein Protein  
The output is a structure file containing the protein embedded in the membrane. If a topology
file is provided, the number of lipid and
solvent molecules will be updated to match the new structure file.  
For a more extensive manual see Wolf et al, J Comp Chem 31 (2010) 2169-2174, Appendix.

SHORT METHOD DESCRIPTION  
------------------------  
1. The protein is resized around its center of mass by a factor -xy in the xy-plane
(the membrane plane) and a factor -z in the z-direction (if the size of the
protein in the z-direction is the same or smaller than the width of the membrane, a
-z value larger than 1 can prevent that the protein will be enveloped by the lipids).  
2. All lipid and solvent molecules overlapping with the resized protein are removed. All
intraprotein interactions are turned off to prevent numerical issues for small values of -xy
or -z  
3. One md step is performed.  
4. The resize factor (-xy or -z) is incremented by a small amount ((1-xy)/nxy or (1-z)/nz) and the
protein is resized again around its center of mass. The resize factor for the xy-plane
is incremented first. The resize factor for the z-direction is not changed until the -xy factor
is 1 (thus after -nxy iterations).  
5. Repeat step 3 and 4 until the protein reaches its original size (-nxy + -nz iterations).  
For a more extensive method description see Wolf et al, J Comp Chem, 31 (2010) 2169-2174.

NOTE  
----  
- Protein can be any molecule you want to insert in the membrane.  
- It is recommended to perform a short equilibration run after the embedding
(see Wolf et al, J Comp Chem 31 (2010) 2169-2174), to re-equilibrate the membrane. Clearly
protein equilibration might require longer.

### Files

| option | filename | type | description |
| --- | --- | --- | --- |
| **-f** | into\_mem.tpr | Input | Run input file: tpr tpb tpa |
| **-n** | index.ndx | Input, Opt. | Index file |
| **-p** | topol.top | In/Out, Opt. | Topology file |
| **-o** | traj.trr | Output | Full precision trajectory: trr trj cpt |
| **-x** | traj.xtc | Output, Opt. | Compressed trajectory (portable xdr format) |
| **-c** | membedded.gro | Output | Structure file: gro g96 pdb etc. |
| **-e** | ener.edr | Output | Energy file |
| **-dat** | membed.dat | Output | Generic data file |

### Other options

| option | type | default | description |
| --- | --- | --- | --- |
| **-[no]h** | bool | no | Print help info and quit || **-[no]version** | bool | no | Print version info and quit || **-nice** | int | 0 | Set the nicelevel || **-xyinit** | real | 0.5 | Resize factor for the protein in the xy dimension before starting embedding || **-xyend** | real | 1 | Final resize factor in the xy dimension || **-zinit** | real | 1 | Resize factor for the protein in the z dimension before starting embedding || **-zend** | real | 1 | Final resize faction in the z dimension || **-nxy** | int | 1000 | Number of iteration for the xy dimension || **-nz** | int | 0 | Number of iterations for the z dimension || **-rad** | real | 0.22 | Probe radius to check for overlap between the group to embed and the membrane || **-pieces** | int | 1 | Perform piecewise resize. Select parts of the group to insert and resize these with respect to their own geometrical center. || **-[no]asymmetry** | bool | no | Allow asymmetric insertion, i.e. the number of lipids removed from the upper and lower leaflet will not be checked. || **-ndiff** | int | 0 | Number of lipids that will additionally be removed from the lower (negative number) or upper (positive number) membrane leaflet. || **-maxwarn** | int | 0 | Maximum number of warning allowed || **-[no]start** | bool | no | Call mdrun with membed options || **-[no]v** | bool | no | Be loud and noisy || **-mdrun\_path** | string |  | Path to the mdrun executable compiled with this g\_membed version |

---

http://www.gromacs.org  
gromacs@gromacs.org
